# The Effect of Coherence on Instruction Following from News Outlets

**DOI:** 10.3390/bs16010102

**Published:** 2026-01-12

**Authors:** Michael O’Sullivan, Conor McCloskey, Louise McHugh

**Affiliations:** School of Psychology, University College Dublin, D04 V1W8 Dublin, Ireland; conor.mccloskey1@ucd.ie (C.M.);

**Keywords:** relational frame theory, coherence, rule-governed behavior, rule-following, news media

## Abstract

Research has shown that preferences exist in following information from coherent sources and that incoherent material can diminish overall trust in sources from readers. In line with relational frame theory, coherence, or “sense-making”, has emerged as an important factor in the process of rule-following, but some research has been confounded by the degree of extremity used to establish coherence. The present study investigated the role of speaker coherence in rule-following preferences between newspaper outlets. Participants (N = 64) each viewed four news articles that had been published across two anonymized Irish newspaper outlets. Each article was categorized by its level of coherence and level of controversy. Rule-following was measured through the likelihood of participants adopting new habits and following general advice from the news outlet after reading each story. Participants also selected their preferred outlet from which to follow general advice at the end of the study. Results indicated that participants had greater rule-following preferences for coherent outlets, regardless of how controversial the material was perceived to be. Speaker coherence was discussed in terms of its impact on the perceived credibility of media outlets and avenues for reducing polarization.

## 1. Introduction

Rule-governed behavior (RGB) is an area of key importance in behavioral accounts of human action, with Skinner’s (1966) original research on RGB establishing that rules permit learning without having to directly engage with reinforcement strategies. Relational frame theory (RFT; Hayes et al., 2001) is an account of human language and cognition grounded in behavior analysis that stipulates that complex human cognition is a result of an ability to create and derive relationships between stimuli arbitrarily, providing a framework to explain the formation of stimulus relations (Bordieri et al., 2015). RFT has facilitated an account of rule-governed behavior, conceptualized as behavior under the control of a verbal antecedent (a rule, either self-generated or provided by a “speaker”) and an implied source of reinforcement (typically consequences stated within the rule that are likely to be experienced should the rule be followed). Research on RGB has explored the conditions under which rules may or may not be followed (Stapleton, 2020; Törneke et al., 2008).

In line with RFT, coherence is a characteristic of patterns of relational responding, with coherence referring to the extent to which a particular pattern is consistent with previously established patterns of responding (Harte et al., 2020). Coherence is one of the core dimensions proposed within the hyperdimensional multilevel framework (HDML), a framework for the analysis of relational responding in individual cases (relational responding refers to the behavior of responding to stimuli in terms of another stimulus; for a discussion of coherence as it is linked to relational responding, see Bern et al., 2021), and on a higher level, the term coherence has been used to refer to a form of sense-making that can function as a reinforcer for relational responding (Barnes-Holmes et al., 2001; Wray et al., 2017). Bianchi et al. (2021) examined coherence by exploring preference in rule-following, as it relates to the speakers (or rule-givers) who were experimentally manipulated to show higher or lower levels of coherence in their initial speech. This experiment found that participants persisted in following instructions given by a coherent speaker, using the information of these speakers as a trusted source of information in a points-based task.

A study by Zhang (2023), based in the United States, found that by publishing a political endorsement, multidisciplinary scientific journal *Nature* negatively affected its perception as an unbiased and informed source in the eyes of some readers. The endorsement in question was an editorial endorsement in favor of presidential candidate Joe Biden during the 2020 United States presidential election. This experiment determined that while the political endorsement did not affect Biden supporters’ opinion of *Nature*, supporters of opposing candidate Donald Trump who viewed the article subsequently rated *Nature* as being significantly less impartial and less knowledgeable on science-related issues faced by society. Zhang (2023) argues that by publishing a story incongruent with some readers’ personal beliefs, *Nature* had jeopardized its reputation and risked some future readers disregarding advice they may publish.

Both the study by Bianchi et al. (2021) and Zhang (2023) explore aspects of decision-making related to perceived coherence in prior material. In interpreting Zhang’s (2023) study in line with RFT, an account in line with the interpretation presented in the original study would indicate that the political endorsement given was perceived by participants as being low in coherence, leading to a generalized reduction in the willingness of these participants to follow rules associated with this source in the future. While this interpretation aligns to a degree with the study by Bianchi et al. (2021), a contrary interpretation of these findings exist, in which the effect on speaker preference was an expression of ingroup loyalty and hostility to others who do not share the same views (hereafter referred to as polarization; Hart et al., 2009; Iyengar & Westwood, 2015; Wolfe & Britt, 2008); it is possible that participants, who were not recruited from scientific professions, may have viewed the journal endorsement as evidence of political bias in the journal that runs contrary to their own political views. The present study seeks to explore the role of coherence in decision-making while considering the factor of polarization in this process. In doing this, this study will explore how coherence influences intention to follow rules from a speaker through testing reactions to outlets that are established as high or low in coherence. Simultaneously, this study will explore the possibility that preference is linked to polarization through controlling the perceived controversy of material displayed by outlets. 

## 2. Methods

### 2.1. Pilot Study

A pilot study was conducted with 54 participants (age range of 18–64, *M* = 26.52, *SD* = 9.85) to determine which of the selected news articles were seen as controversial and coherent. The pilot study was advertised through social media posts (LinkedIn, X, Instagram) and a student mailing list. Four classifications for news articles were established: “high-controversy/high-coherence”, “high-controversy/low-coherence”, “low-controversy/high-coherence”, and “low-controversy/low-coherence”.

In completing the study pilot, participants read 10 articles that had been previously published within the past 18 months by two news outlets (The Irish Times, a broadsheet newspaper, and the Irish Mirror, a tabloid newspaper). The researchers selected articles concerning topics likely to elicit a high/low-controversy response (e.g., immigration, gender identity). The articles presented included a headline, subheading, photo, and shortened article body. To prevent any pre-existing biases towards either newspaper, they were referred to as Newspaper A and Newspaper B in respective order. After each article, participants were asked to rate how familiar they were with the story’s content, from “very familiar” to “not familiar at all”. They then rated how controversial they found the article on a four-point Likert scale, ranging from “highly controversial” to “not controversial at all”. For the coherence rating, participants were asked, “How closely does this news story align with your previous understanding of the issue presented in this article?” rating their response on a five-point Likert scale ranging from “very closely” to “very differently”. Upon completion, the source of each article was disclosed to participants for debrief.

### 2.2. Participants

For the primary study, 64 participants with an age range from 21 to 70 (*M* = 35.40, *SD* = 11.07) were recruited. Participants were recruited locally through advertisements on social media, student mailing lists, and through an online participation platform. The required criteria for participation were for participants to be at least 18 years of age and ordinarily resident in Ireland. Participants also indicated their political preferences (liberal = 49.2%, politically neutral = 40%, conservative = 9.2%). 60 participants were retained at the follow-up data collection phase.

### 2.3. Selected News Articles

The primary study was completed using Google Forms. Results from the pilot study were used to select the articles for the primary study, with comparisons of participant-rated controversy and coherence scores used to select four articles that fit into the high/low-coherence and high/low-controversy coherence categories. Stories from Paper A were always high in coherence (despite differences in controversy levels), making Paper A the high-coherence source and Paper B the low-coherence source. This categorization is listed in Table 1 below.

Outlets were again presented to participants as Newspaper A and Newspaper B. The news stories were presented to participants as a headline, a subheading, an image attached to the news story, and the article body. To reduce the time needed to complete the survey, with the view of improving participant retention in the follow-up survey, the article body for each story was cut to only include approximately 100 words.

### 2.4. Procedure

Ethical approval was granted by the [REDACTED FOR MASKED REVIEW] Human Subjects Ethics Committee prior to the commencement of the pilot study. Participants provided consent to participate after viewing an information sheet outlining the background to the study. To allow for an anonymized follow-up survey, each participant provided a unique ID code with which the researchers could match their initial responses to the follow-up survey. Demographic information was then collected from participants.

Participants were presented with each of the four selected news articles (see Appendix A). Participants first responded to “How closely does this story align with your previous understanding of the issue presented in this article?” on a five-point Likert scale. The following questions measured perceived levels of controversy, using the same scale as in the pilot study, and accuracy of the reporting in the article. To measure coherence, participants were asked to report the degree to which the information presented in Newspaper A/B aligned with their general worldview, and participants were further asked to rate their likelihood of following advice and behaviors outlined in either source. After viewing all four news articles, participants were asked to choose a preference between following advice from Newspaper A, Newspaper B, or neither, should conflicting information be printed in each newspaper. After indicating their preference, participants had the opportunity to provide a brief explanation behind their answers.

#### Follow-Up Survey

All participants were contacted one week later with an invitation to participate in a brief follow-up survey. The purpose of this follow-up was to investigate any lasting effects of the high/low-coherence stories and if participants would be more willing to follow advice published in Paper A/B. Participants were presented with a brief description of the survey and provided consent to their responses being recorded.

Participants were reminded of the articles they were presented with during the initial survey. Participants were first shown the headline and subheading from each article published in Paper A. The two measures of rule-following preference were reused: “How likely would you be to follow general advice that appears printed in Newspaper A?” and “How likely would you be to adopt new habits if articles in Newspaper A suggested that there would be benefits to doing so?”. After completing this section, participants were shown the headline and subheading from each article published in Paper B. Participants then responded to the same two rule-following questions. Finally, participants were asked to again indicate their preference for following advice or adopting new habits between Paper A and B. Finally, participants were fully debriefed at the end of the survey. All sources and original article links were disclosed to candidates.

## 3. Results

### 3.1. Rule-Following Preferences

A repeated-measures ANOVA indicated that rule-following preference differed significantly between the four stories (*F* (3, 180) = 18.46, *p* < 0.001, η_p_^2^ = 0.24).

A post hoc pairwise comparison, using the Bonferroni correction, specified that Story 1 and Story 2 differed significantly (2.89 vs. 3.8, respectively, *p* < 0.001), as did Story 1 and Story 4 (2.89 vs. 3.37, *p* < 0.001). Story 2 differed significantly in score with all other stories; in addition to differing from Story 1, Story 2 differed significantly from Story 3 (3.8 vs. 3.08, *p* < 0.001) as well as Story 4 (3.8 vs. 3.37, *p* = 0.005).

Rule-following preference between sources is presented in Table 2 below, with higher scores indicating stronger intentions to follow instructions from these sources.

### 3.2. Initial Newspaper Preference vs. Follow-Up

Preference for news outlets between initial data collection and follow-up is displayed in Table 3 below.

## 4. Discussion

This study examined speaker coherence and perceived controversy in relation to media publications and news articles. The researchers set out to test how an article’s level of coherence can impact speaker (or in this case, newspaper) preference and receptivity to rule-following within a general population sample. Using a repeated measures ANOVA, significant differences were observed in rule-following ratings between the high- and low-coherence news articles. Participants reported greater rule-following preferences for the high-coherence stories published in Paper A compared with the low-coherence stories, with 50% (initial survey) and 55% (follow-up survey) of participants choosing Paper A as the outlet that they would elect to follow general advice from.

Participants displayed a preference to follow advice from the outlet in which the coherent stories were published, regardless of whether the stories were perceived as controversial. Consistent with this outcome was that participants reported significantly lower preferences for rule-following while reading the low-coherence stories published in Paper B. Moreover, Paper B saw the smallest percentage (10% initial survey, 16.7% follow-up) of participants choosing to follow general advice from said publication on future topics, suggesting that messages that show less alignment with the learning histories of readers can impact on the receptivity of readers in the future.

These results expand upon the findings in Zhang’s (2023) study, which examined how a political endorsement affected *Nature’s* credibility as an informed and impartial outlet, finding that endorsements served to reduce *Nature’s* credibility among readers who found it incoherent with their worldview and did not positively impact its credibility among readers who already agreed with the article. In light of the present findings, which suggest that preference is traceable to coherence rather than controversy or political polarization, Zhang’s (2023) results can be interpreted in part as a function of relational coherence. The present findings imply that the effects shown in the *Nature* study are not all attributable solely to the level of polarization. This outcome presents opportunities for future research to investigate the process of political polarization, accounting for how coherence may create a preference for certain narratives.

Bianchi et al. (2021) recommended that more avenues for measuring coherence be explored. The experiments carried out by Harte et al. (2020, 2021) relied on manipulating coherence through the use of feedback and reversed reinforcement contingencies. The present study manipulated coherence through the content of the information provided. By altering details within articles and misattributing quotations, the researchers sought to reduce participants’ ratings of coherence for the story. As such, the researchers’ measure of coherence (participants rating how closely the news article fit with their general worldview) was supported by Story 2 receiving the least favorable rating for coherence out of all the articles. This result suggests that this measure adequately captures the operation of coherence, as the stories selected by the researchers to fit each high-/low-coherence category received the predicted coherence ratings from participants.

The present study was not without limitations. A better representation of contrasting political views will be important for future research surrounding coherence as it relates to news outlets and public information. Participants were not pre-screened for political preferences before receiving access to the study, with a high percentage (49.2%) falling on the “liberal” side of the political spectrum. In contrast, only 9.2% self-identified as “conservative”, with the remaining 40% choosing “politically neutral”. It is possible that some of these percentages may not accurately represent political ideologies in the sample. Self-identification on the political spectrum can be difficult to control for, as political labels often digress from actual ideologies. In the US, “conservative” has been argued to now represent more of an identity than a robust set of political ideals (Claassen et al., 2015). Given the prominence of the US political sphere in Irish news, some participants in the present study may not have wished to associate themselves with this conservative identity. It would be beneficial for future research to obtain a more balanced sample that reflected more varied political views, taking any tendencies in participants to separate political ideology from a political identity into account.

This study selected two newspaper formats from which to source articles: a broadsheet and a tabloid. Although only a brief portion of each original article was presented to participants, ancillary characteristics of articles presented may have appealed to existing preferences towards either broadsheets or tabloids. Future research aiming to recreate this study could therefore account for this through testing a wider selection of articles, allowing for an equal balance of high/low-coherence and high/low-controversy stories across sources. Additionally, the stories were presented in a fixed order, creating a risk of an order effect. This issue could be rectified in future studies through the use of a survey platform that allows for the randomization of the presentation of materials. Additionally, this study required participants to provide self-report data when establishing preferences between outlets. Future research may benefit from the use of behavioral measures in establishing preference.

Coherence has a demonstrated effect on a newspaper’s ability to provide advice and information. Regardless of whether the reader views a story as polarizing or non-polarizing, the extent to which it “makes sense” with their worldview is what impacts their receptivity to adopting new behaviors advised by the outlet. The complexities of how particular narratives and speakers propagate have meaningful implications for ensuring collective adherence to public information published in news outlets, such as public health information and advice. Future research should utilize a sample containing a wider variety of political opinions and, likewise, test a wider range of polarizing/non-polarizing stories.

In conclusion, this study explored the role of coherence and controversy in relation to perception of sources and intentions to follow information in such sources in the future. In building on research highlighting the role of sense-making in preference for rule-following regarding sets of speakers, the present results broadly support the existence of a preference for coherent speakers, supporting the literature on relational coherence, which was central to the development of this study, while also supporting previous findings that distrust of sources can stem from incoherent material distributed from such sources, suggesting that this remains the case regardless of the level of controversy present within material.

## Figures and Tables

**Table 1:** Story Categorization based on Pilot Results.

	High-Controversy	Low-Controversy
High-coherence	Story 1 (Paper A): *Sharp division over teaching of gender identity and use of pronouns in updated sex education syllabus*	Story 3 (Paper A): *Dublin Metro hearings resume after 15 years as first trains may run by mid-2030s*
Low-coherence	Story 4 (Paper B): *Pupils in Dublin primary school told to refer to their gender-neutral teacher as ‘they’ in the classroom*	Story 2 (Paper B): *Taoiseach says Fine Gael ‘being out of office would not be good for Ireland’*

**Table 2:** Mean Scores and Standard Deviation for Rule-Following Preference.

Story	*M*	*SD*
Story 1 (high coherence/high controversy)	2.89	0.91
Story 2 (low coherence/low controversy)	3.80	0.87
Story 3 (high coherence/low controversy)	3.08	0.74
Story 4 (low coherence/high controversy)	3.37	0.93

Note: lower scores are indicative of greater rule-following preferences.

**Table 3:** Frequency Choices between Papers A and B.

Source	Initial Survey	Follow-Up Survey
Newspaper A	30 (50%)	33 (55%)
Newspaper B	6 (10%)	10 (16.7%)
Neither	24 (40%)	17 (28.3%)

## Data Availability

Data from this study is available upon reasonable request.

## Appendix A. Articles Presented to Participants

Story one:

**Sharp division over teaching of gender identity and use of pronouns in updated sex education syllabus**

A number of Catholic bodies, while welcoming the move to update the curriculum, said the ethos of schools must be respected in delivering the program

Sharp divisions over gender identity and the use of pronouns in schools are revealed in submissions to the State’s advisory body on the curriculum which is updating how sex education is taught.

The National Council for Curriculum and Assessment (NCCA) is finalizing a new Social Personal and Health Education (SPHE) curriculum, which will provide 100 h of learning over the three-year Junior Cycle for 12–15 year olds. It will, from next September, address issues such as gender identity, pornography and sexual consent.

Story two:

**Taoiseach says Fine Gael ‘being out of office would not be good for Ireland’**

Taoiseach Simon Harris has denied that Fine Gael is destined for opposition following the next election.

Fine Gael “being out of office would not be good for Ireland” Taoiseach Simon Harris has claimed as he ruled out going into opposition in the next election.

As he confirmed that he sent Sinn Féin President Mary Lou McDonald flowers following her hysterectomy, he once again insisted he would not go into power with her party.

In a veiled dig at Sinn Féin, he also branded it “trashy” when TDs tweet pictures stating that they are returning part of their salary to the State.

Story three:

**Dublin Metro hearings resume after 15 years as first trains may run by mid-2030s**

More than 120 parties to speak at planning meetings on long-delayed underground line including affected residents and campaign groups

It is 15 years since An Bord Pleanála last opened a planning hearing into a metro rail line for Dublin, 14 years since it granted permission for Metro North, 13 years since the Government shelved it, nine years since it announced it was back on track, five years since the preferred route was confirmed, and a year and a half since Transport Infrastructure Ireland (TII) lodged its application for the line.

On Monday morning, a new An Bord Pleanála metro hearing will open, and Dubliners and visitors may now have to wait just a little more than a decade for the first underground train to chug down the line.

Story four:

**Pupils in Dublin primary school told to refer to their gender-neutral teacher as ‘they’ in the classroom**

The children from the Dublin Educate Together school—aged 8 and 9—were told this on their first day back, according to parents

Kids in a primary school have been told to refer to their gender-neutral teacher as “they” in the classroom. The Third Class pupils returned to school last week to be informed their new teacher wants to be referred to by first name only, or the pronoun “they”.

The children from the Dublin Educate Together school—aged 8 and 9—were told this on their first day back, according to parents. They have since been corrected by the teacher for saying “she” and taught to say “they” instead.

It is believed to be the first time a school in Ireland has moved to accommodate a teacher’s gender identity within the classroom.
